# Prolonged anxiety on habituation of the cold shock response

**DOI:** 10.1186/2046-7648-4-S1-A131

**Published:** 2015-09-14

**Authors:** Heather C Massey, Jo Corbett, Christopher Wagstaff, Michael J Tipton, Martin Barwood

**Affiliations:** 1 Extreme Environments Laboratory, Department of Sport and Exercise Science, University of Portsmouth, UK; 2 Department of Sport, Exercise and Rehabilitation, Northumbria University, UK

## Introduction

Variation in the cold shock response (CSR) can be explained by physiological factors [1], habituation [2]; and possibly "psychological" influences. Acute anxiety on cold-water immersion (CWI) increases the magnitude of the CSR in unhabituated volunteers and eliminates the reduction in the response seen after habituation [3]. Recently it was demonstrated that habituation of the CSR includes a significant perceptual component [4]. When the threat of CWI scenario was reduced, anxiety associated with being immersed was also reduced. In contrast, prolonged anxiety during repeat CWIs may prevent habituation. Therefore, it was hypothesized that prolonged anxiety reduces the extent of CSR habituation.

## Methods

Sixteen volunteers (females *n *= 4, male *n *= 12) gave their consent to participate in this ethically approved study. Volunteers completed seven, 7 min CWIs (water temperature, 15 °C) on consecutive days. ECG, ventilatory and anxiety responses were measured. CWI 1 was a control immersion (CON1), prior to the 2^nd ^to 5^th ^CWI, volunteers performed three min mental maths tests and were told incorrect answers would extend their immersion time. They were also told the water would be a degree colder on each immersion; it remained unchanged. One of the final two immersions (CON2) was a repeat of CON1, and the other was the final maths test with the same conditions as immersion five (MATHS). Upon completion, all volunteers were fully debriefed and gave their consent to release the data retrospectively. Two way repeated measures ANOVA analysis were performed using the data.

## Results

There were no differences between CON1, 2 or MATHS in the heart rate, ventilatory or anxiety responses to CWI (Table 1). In contrast, there were main effects of time, with all variables increasing from baseline during the first minute of CWI and then reducing.

## Discussion

The present data suggest the habituation of the physiological responses commonly seen with repeated CWI can be inhibited by chronic anxiety throughout CWI; the hypothesis is accepted. This confirms and progresses the work of Barwood *et al *(2014), indicating that maintenance of anxiety during repeated CWIs inhibits habituation.

**Table 1:** Mean (standard deviation) heart rate, inspired volume and anxiety variables during cold water immersions (CWI).

	Heart Rate (beats.min^-1^)			Inspired Volume (L.min^-1^)			Anxiety		
	CON 1	CON 2	MATHS	CON 1	CON 2	MATHS	CON 1	CON 2	MATHS

Baseline	84(14)	81(13)	80(13)	15.9(7.8)	15.7(6.1)	13.5(6.6)	5(5)	4(4)	6(5)

CWI min 1	98(18)*	92(15)*	95(15)*	47.8(28.9)*	43.1(17.1)*	43.1(20.4)*	8(6)*	6(5)*	9(6)*

CWI min 2	89(16)	82(12)	88(17)	37.9(22.0)	31.6(16.3)	34.1(18.8)			

CWI min 3	85(15)	80(13)	84(15)	32.4(20.7)	24.7(11.8)	27.0(13.8)	6(4)	5(5)	6(5)

CWI min 7	84(16)	74(12)	76(13)	25.2(21.1)	17.9(6.7)	22.7(11.7)	4(4)	4(4)	5(5)

Anxiety 20 = extremely anxious, 0 = not anxious, * = different from Baseline (*P *< 0.05)
